# Age, Gender and Suicidal Ideation Following Voluntary HIV Counseling and Testing

**DOI:** 10.3390/ijerph9020521

**Published:** 2012-02-10

**Authors:** Lourens Schlebusch, Romona Devi Govender

**Affiliations:** 1 Department of Behavioural Medicine, Nelson R Mandela School of Medicine, University of KwaZulu-Natal, Durban, 4001, South Africa; 2 Department of Family Medicine, Nelson R Mandela School of Medicine, University of KwaZulu-Natal, Durban, 4001, South Africa; Email: govenderr1@ukzn.ac.za

**Keywords:** age, gender, HIV-test results, suicidal ideation, voluntary counseling and testing

## Abstract

The aim of this study was to determine the prevalence of suicidal ideation in patients who were tested for HIV-infection and whether along with their HIV status, age and gender influenced their risk for suicidal ideation. The sample consisted of 189 patients who attended a voluntary HIV counseling and testing clinic (VCT) at a general state hospital in Durban, South Africa. Their mean age at baseline was 34.2 years, with an age range of between 16–79 years. Seropositivity, age and gender were significantly associated with suicidal ideation. The majority of these patients were in the younger age group, and young males had a 1.8 times higher risk for suicidal ideation than females. Although risk factors for seropositive-related suicidal ideation can be complex and multi-factorial, this study identified a young age and male gender as important high risk factors in the sample studied. It is recommended that all, but especially young male HIV-infected patients seen at a VCT clinic be screened for suicidal ideation and that early intervention to prevent subsequent suicides or suicidal attempts be included in pre- and post-test HIV counseling.

## 1. Introduction

Although there are geographic variations between and within certain countries and regions, suicidal behaviour and HIV/AIDS continue to be major global health care priorities [1,2]. In South Africa, many suicides and attempted suicides go unreported, but available statistics are alarming, with a prevalence rate of between 17–25 per 100,000 of the population and a suicide to attempted suicide ratio of about 1:20 [3,4,5,6]. Suicide accounts for about 9.5% of non-natural deaths in young people and 11% in adults in the country, with the average age of suicide being 35 years and for suicide attempts 20–29 years followed by the 10–19 year age group [7,8]. 

This situation is compounded by the HIV/AIDS pandemic. Nearly three decades after its initial diagnosis, HIV/AIDS is still a devastating and debilitating disease, especially in developing countries. Sub-Saharan Africa has one of the highest global prevalence rates of HIV/AIDS, accounting worldwide for 67% of current HIV-infections, 68% and 91% for new infections among adults and children respectively, and 72% of AIDS-related deaths [2]. Women and youth continue to be affected disproportionately [2]. South Africa has one of the biggest seropositive populations where some 5.7 million people are living with HIV/AIDS, although the country has one of the biggest antiretroviral therapy programmes in the world, which has produced substantial health benefits and various major preventative strategies [2]. Heterosexual exposure is the main mode of transmission. Despite the fact that more than 85% of South African youth have been exposed to multi-media HIV education and awareness campaigns, infection rates among both the youth and females remain high [9]. The epidemic differs considerably between provinces, but the province of KwaZulu-Natal (where the present study was done) has one of the largest provincial prevalence rates (39% or more of the population) and up to one third of patients seen in some ante-natal clinics are HIV-positive [2,4,10,11,12].

Earlier studies reported higher suicide risks in HIV/AIDS-infected people compared to the general population, but in some instances this seems to have improved with better treatment [4,12,13,14]. Figures, however, continue to vary. For example, although few published studies are available in the rest of Africa, South African studies have found that people suffering from HIV/AIDS have an increased suicide risk [4,12,15,16,17,18]. Likewise, studies on suicidal ideation in the general population in South Africa have produced diverse results. These range from 4% in young primary school children to 24% or higher in high school students and adolescents to 9.1% or higher in adults [4,7,19]. In a community survey in the port city of Durban, situated in the province of KwaZulu-Natal with its high HIV-infection rates, suicidal ideation was found to be as high as 25.4% [20]. 

Researchers have identified different patterns of increased risk for suicidal behaviour through the progression of HIV/AIDS, which are associated with, *inter alia*, the manner in which HIV testing is done; inadequate psychosocial support at the time; anxiety before the results are known; being diagnosed seropositive; the development of full-blown AIDS with its implications; later stages of the disease which may result in a decrease of quality of life characterized by physical and mental deterioration; sufferers who want to control the way in which they die, thus considering suicide as an option; and the vulnerability of survivors whose loved ones have died as a result of HIV/AIDS-related suicide, especially if they themselves are also seropositive [4,12,18]. Although there are some earlier studies done elsewhere on mental health states and suicidal ideation in acute HIV-infection [21,22,23] and some [22] have shown that within the context of confidential HIV-testing and extensive pre-post counseling in over 15% of both seropositive and seronegative patients suicidal ideation can persist, there is a dearth of similar research in Africa. In this regard, in a previous South African study we reported a significant presence of suicidal ideation and clinical depression in seropositive patients who underwent voluntary HIV-testing [15]. However, despite the high South African suicidal behaviour and HIV/AIDS prevalence rates in the young, suicidal ideation in relation to age, gender and seropositivity at the time of testing for HIV-infection, has not been well-researched. In addition, studies are sparse on suicidal ideation and gender differences in HIV-positive persons, and findings are inconclusive [1,12,22]. One study [12] reported no gender differences in mood disorders which accounted for the most common psychiatric diagnosis in HIV-related suicidal behaviour. Therefore, as part of our ongoing research on HIV/AIDS-related suicidal behaviour, the aim of the present study was to determine the prevalence of suicidal ideation in patients tested for HIV-infection and whether along with their HIV status, age and gender influenced their risk of suicidal ideation. 

## 2. Methodology

### 2.1. Sample and Procedure

The sample recruited consisted of 189 patients who were referred by various health professionals to an HIV Voluntary Counselling and Testing (VCT) clinic at a general state hospital in Durban, South Africa over a three-month period. The mean age of the initial sample at baseline was 34.2 years (SD = 10.7) with an age range of between 16–79 years. Of these, 157 (83.1%) tested HIV positive. The study was ethically approved and permission to conduct it was granted by the health institution. The patients entered into the study voluntarily and signed informed consent and received relevant pre-and post-test counseling. All those who did it were offered ARV treatment.

### 2.2. Measures

A research-designed questionnaire was used to record socio-demographic data, and patients were advised not to answer any questions that they deemed too personal and/or too sensitive if they so wished. In line with other studies [15,24,25] suicidal ideation was measured using the Beck Hopelessness Scale (BHS) and item 9 of the Beck Depression Inventory (BDI). These were administered to each patient at two time points, *viz*.: at 72 hours and again at six weeks following testing and after being advised of their HIV status. The time frame selected was approximately consistent with that reported before in studies on suicidal ideation and testing for HIV [21,23,26]. The BHS and BDI were both translated into isiZulu and then back translated with the assistance of a professional translator in order to accommodate the isiZulu speaking patients in the sample (who constituted the largest African ethnic group). A cut-off score of 9 or above on the BHS [27] along with item 9 on the BDI were used to measure suicidal ideation. Item 9 of the BDI deals specifically with thoughts of committing suicide, and patients endorsed 0 (“I don’t have any thoughts of killing myself”), 1 (“I have thoughts of killing myself, but I would not carry them out”), 2 (“I would like to kill myself”), or 3 (“I would kill myself if I had the chance”). As in previous research for the purpose of the present study, “suicidal ideation” was defined as a score of 1 to 3 [15,22].

### 2.3. Data Analyses

The SPSS version 15.0 was used to analyse the data. Pearson’s Chi Square was used to perform univariate analyses, and a generalized linear regression modeling for the binomial family (binreg) was used to assess independent risks for suicidal ideation. A log link was specified to estimate risk ratios, while a backwards selection method was used to fit a model with only statistically significant independent variables. This model was used (in preference to the logistic regression model) because (according to the SPSS manuals) it expands the general linear model so that the dependent variable islinearly related to the factors and covariates via a specified link function and the model allows for the dependent variable to have a non-normal distribution and covers widely used statistical models, through its very general model formulation. It is, however, equally allowed to predict a binary variable with both methods.

## 3. Results and Discussion

### 3.1. Results

Because some patients from the original sample were lost on follow-up, and because of incomplete data for some patients, totals reflected in the text and tables vary. Also from a socio-demographic point of view, the seronegative patients as a group generally had higher educational levels than the seronegative patients and more were employed and in stable partner relationships. These variable, however, were not separately analysed in the present study.

**Table 1:** Suicidal ideation at 72 hours by HIV status.

			Suicidal ideation		Total
			no	yes	
**HIV-test result**	negative	Count	30	0	30
		Row %	100.0%	0%	100.0%
	positive	Count	124	32	156
		Row %	79.5%	20.5%	100.0%
Total		Count	154	32	186
		Row %	82.8%	17.2%	100.0%

Pearson’s Chi Square = 7.433, *p* = 0.006.

**Table 2:** Suicidal ideation at 6 weeks by HIV status.

			Suicidal ideation		Total
			no	yes	
**HIV-test result**	negative	Count	21	0	21
		Row %	100.0%	0%	100.0%
	positive	Count	79	32	111
		Row %	71.2%	28.8%	100.0%
Total		Count	100	32	132
		Row %	75.8%	24.2%	100.0%

Pearson’s Chi Square = 7.991, *p* = 0.005.

Table 1 and Table 2 show that none of the HIV-negative patients displayed suicidal ideation after being informed of their seronegative status. However, at both 72 hours and at 6 weeks post HIV-testing, there was a significant association between HIV-positive test results and suicidal ideation. At 72 hours the risk of suicidal ideation in those who tested HIV positive increased statistically significantly from 20.5% to 28.8% at 6 weeks as indicated in the relevant tables. 

Age was a significant risk factor for the presence of suicidal ideation in the seropositive patients at both time points, that is at 72 hours (RR = 1.031; Std. Err =0.039; z = 2.32; *p* = 0.020; 95% CI = 1.004 to 1.059) and 6 weeks (RR = 1.050; Std. Err =0.006; z = 7.78; *p* = 0.001; 95 % CI = 1.037 to 1.063). In this group, at 6 weeks both age and gender were significantly associated with suicidal ideation in seropositive patients (Table 3). Despite the fact that a wide age range was represented in the sample cohort, the majority of the seropositive patients with suicidal ideation fell within the younger age group (<30) which is consistent with the age-related spread of the disease and the increase in suicidal behaviour in younger people. Males had a 1.8 times higher risk of suicidal ideation than females (*p* = 0.025). 

**Table 3:** Suicidal ideation at 6 weeks by Age and Gender.

	Risk Ratio	Std. Err.	z	*P* > |z|	95% CI
Age	1.03143	0.00722	4.42	0.000	1.017376 to 1.045678
Gender	1.783475	0.4616398	2.24	0.025	1.07384 to 2.962064

### 3.2. Discussion

People react differently to a potentially life-threatening disease in which hopelessness and a sense of loss of control often correlate with suicidal ideation [4,12,14,28,29]. Also, suicidal risk factors associated with HIV/AIDS and HIV-testing are complex and multifactorial [4,12,15,22,23,30,31]. Common co-morbid factors in HIV/AIDS-related suicidal behaviour include amongst others: acute stress reactions; adjustment disorders; obsessive compulsive disorders involving obsessive ruminations and scrutiny for disease progression; bereavement reactions; neuropsychological/cognitive impairment; personality disorders; depression; mania; psychoses; substance abuse; pre-existing psychiatric disorders; neuropathology; neuropsychiatric side-effects of ARV and psychotropic medication; anxiety reactions associated with the “worried well”, such as “AIDS phobia”, “AIDS panic” or “AIDS anxiety”; poor social support; fear of disclosure/stigmatization; socio-economic pressures and fear of employment discrimination; as well as relationship problems and the fact that patients often blame their partners for infecting them [4,12,30,32]. The following have also been found to contribute to vulnerability in this regard in some African communities: a failure to meet the psychosocial needs of young people living with HIV/AIDS; a culture of silence related to a fear of being shunned; certain religious leaders who preach that the epidemic is punishment for sexual sin; a disdain towards HIV/AIDS prevention messages and safe sex; and retrogressive cultural beliefs blaming supernatural elements or witchcraft which are compounded by feelings that death is inevitable [4,12,33,34,35]. Additionally, negativity that pervades cognitive processes can be associated with symptoms of depression following HIV-testing in a developing society such as the one where the study was done, where HIV-infected patients often see their situation as overwhelming, filled with obstacles and as a result develop a negative view of their future [4,12,15,18,36]. 

Added to this, are our present findings that support the fact that voluntary HIV-testing associated with a positive test result can be a further risk factor for suicidal ideation, especially in the young in a developing society with its many challenges. Unlike research [22] in a First World context that did not find an increase in suicidal ideation following HIV-testing, we found a significant increase over a 6-week period. Our findings, furthermore, contribute an extra dimension to existing research in the general population which reflects an increase in suicidal behaviour in the younger age groups both in South Africa and elsewhere [1,4,37]. Younger HIV-positive persons have unique stressors which in South Africa have been identified inter alia, as those related to: family disruptions, financial implications, difficulty in accessing health care facilities, meeting the strict criteria for the antiretroviral roll-out programme, and various other psychosocial factors such as partner infidelity and multiple sexual partners [4,12,38,39]. All these factors can serve to further increase susceptibility to suicidal ideation.

Although, seropositive females were also found to be at risk for suicidal ideation, the fact that in the present study males had a higher risk for suicidal ideation is an interesting finding. As in most other parts of the world, more females in South Africa attempt suicide whereas more males commit suicide [4]. However, there is not always a clear association between suicidal ideation and suicide mortality rates [20]. Moreover, suicidal prevalence rates can vary across countries, within populations in specific countries and because of differences in data interpretations [1,40]. Although it is, therefore, difficult to compare data across different studies and cultures, our results of an increased male prevalence rate of suicidal ideation can be explained, in part, by adverse socio-economic considerations and factors such as labour migration which is still a way of life for a large percentage of black males in South Africa. Many of the seropositive patients in our study had lower educational levels, employment difficulties, and partner relationship problems. Males living away from their families because of employment and financial reasons have limited control over their family life, often leading to family breakdown, fragmentation of social networks, increased psychosocial stress, extended sexual networks, and a higher risk of HIV infection among young people who had recently changed their place of residence [4,41]. In addition, the unemployment rate remains high (24% or more in some areas) with a significant percent of young males nationally not being economically active [42]. Competing for limited job opportunities, victimization in the workplace, poverty and debt all have a multiplier effect on the negative emotional impact on young sero-positive males and, therefore, has the potential to increase their risk for suicidal ideation and suicidal behavior [4]. 

### 3.3. Study Limitations

Limitations of the study include the facts that it is preliminary and exploratory and that selective information about the patients were investigated. They were not required to offer any pre-existing psychiatric history, suicidal ideation or a history of suicide attempts. Although this study produced significant findings, the results cannot be axiomatically generalised and require further research.

## 4. Conclusions

This study adds to the current literature on suicidal ideation in the HIV-infected population who attend a VCT clinic, and highlights the role of age and gender and positive HIV-test results in this regard. The hidden nature of suicidal ideation, inaccurate statistics, misclassification on death certification regarding suicide due to confidentiality issues and various other difficulties in the HIV/AIDS population in developing societies, indicate that the situation may be worse than we have found and require further research. Although suicidal ideation is not always a sufficient determinant for suicidal behaviour in its own right, it is an important risk factor that requires early detection. Research has shown that the manner in which a person interprets a negative life experience can influence suicidal ideation, in that an optimistic explanatory style as opposed to a pessimistic one, mitigates the influence of the negative life event and therefore suicidal ideation [43]. In addition to ensuring appropriate access to medical healthcare, it is, therefore, important to increase optimistic cognitions and hopefulness (as opposed to hopelessness) in seropositive patients who attend a VCT clinic. As has been noted before, it is not always the fear itself of a potentially life-threatening disease that act as a suicide risk factor, but how the disease and its sequelae are perceived and managed [12,29]. The high levels of suicidal ideation associated with their positive HIV status in the patients in the present study, confirms the importance of early psychological/psychiatric intervention in these patients. It is recommended that all, but especially young male HIV-infected patients seen at a VCT clinic be screened for suicidal ideation and that early intervention to prevent subsequent suicides or suicidal attempts be included in pre- and post-test HIV counseling. Mental health problems are a significant part of the HIV/AIDS pandemic especially in developing countries where mental health care should be integrated into HIV/AIDS programmes as seen in many developing countries [44]. In this regard, peer mentoring and community involvement constitute innovative strategies that can be used in effective intervention and programme development [5,33,45].
